# Realism of modelled Indian summer monsoon correlation with the tropical Indo-Pacific affects projected monsoon changes

**DOI:** 10.1038/s41598-017-05225-z

**Published:** 2017-07-10

**Authors:** Ziguang Li, Xiaopei Lin, Wenju Cai

**Affiliations:** 1 Physical Oceanography Laboratory/CIMST, Ocean University of China and Qingdao National Laboratory for Marine Science and Technology, Qingdao, 266100 China; 2 CSIRO Oceans and Atmosphere, Aspendale, VIC 3195 Australia

## Abstract

El Niño-Southern Oscillation (ENSO) and the Indian Ocean Dipole (IOD) tend to exert an offsetting impact on Indian summer monsoon rainfall (ISMR), with an El Niño event tending to lower, whereas a positive IOD tending to increase ISMR. Simulation of these relationships in Phase Five of the Coupled Model Intercomparison Project has not been fully assessed, nor is their impact on the response of ISMR to greenhouse warming. Here we show that the majority of models simulate an unrealistic present-day IOD-ISMR correlation due to an overly strong control by ENSO. As such, a positive IOD is associated with an ISMR reduction in the simulated present-day climate. This unrealistic present-day correlation is relevant to future ISMR projection, inducing an underestimation in the projected ISMR increase. Thus uncertainties in ISMR projection can be in part induced by present-day simulation of ENSO, the IOD, their relationship and their rainfall correlations.

## Introduction

Indian summer monsoon rainfall (ISMR) from June to September (JJAS) sources its water from vapour transport by southwesterlies, which supports convection over the Indian subcontinent^1, 2^. Approximately 85% of the annual rainfall total is contributed by ISMR, and year-to-year variations of ISMR often manifest as floods and droughts, causing damage to rain-fed agriculture and affecting the livelihood of more than 1/6^th^ of the world’s population^3–5^. These fluctuations are mainly attributed to slow-varying sea surface temperature (SST) in the tropics^6^. El Niño-Southern Oscillation (ENSO) and the Indian Ocean dipole (IOD), the most prominent air-sea coupled interannual variability over the Indo-Pacific Oceans, are major large-scale drivers of ISMR variability^7, 8^. An El Niño event leads to anomalously low ISMR through the associated Southern Oscillation, suppressing convection over the Indian subcontinent, and vice versa for La Niña^9–11^. By contrast, a positive IOD, when free of an El Niño’s influence, induces atmospheric circulation anomalies which would result in anomalously high ISMR. In particular, the cold anomalies in the eastern Indian Ocean are conducive to vapour transport by generating anomalous meridional circulation^12–14^.

Coupled general circulation models, such as those taking part in Phase Five of the Coupled Model Intercomparison Project (CMIP5), are the useful tools for future climate projections^15^. CMIP5 models project an increase in ISMR in a warmer climate in terms of a multi-model ensemble mean (MMEM) with a reasonably strong inter-model consensus^16, 17^. However, these models suffer from many biases and the projections have a large inter-model spread, undermining the confidence in the projections^17^. One such model bias is the IOD-ISMR positive correlation which is too weak, manifesting as a shorter time fraction in which a positive IOD causes an increase in ISMR, leading to an overly weak IOD-ISMR positive correlation, or an overly strong IOD-ISMR negative correlation^18^. Understanding the cause of this unrealistic correlation is important for tracing the source of uncertainties in future projections of ISMR.

We examine the model performance for the simulated present-day IOD-ISMR relationship through a framework that has been shown to be practicable in regional climate projection^19, 20^. Specifically, we assess whether future ISMR projections are contingent upon the realism of the simulated IOD correlation with ISMR in the present-day climate.

## Results

### Rainfall correlations with the IOD and ENSO in the present-day climate

**Figure 1:**
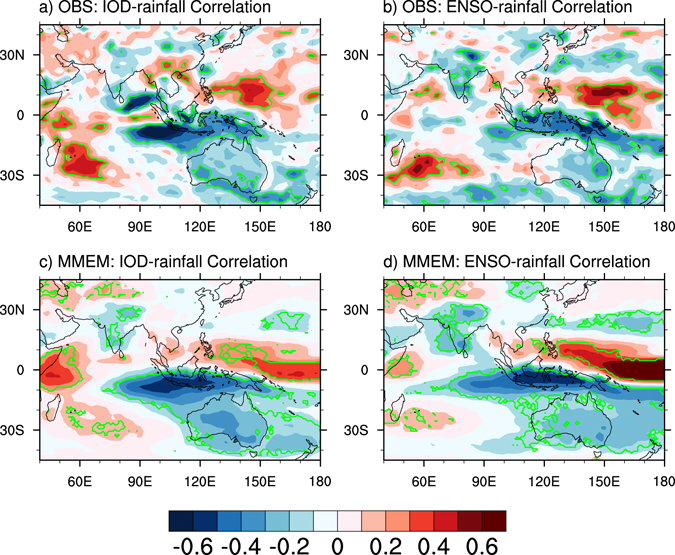
Present-day rainfall’s correlations with ENSO and IOD. (**a**) and (**b**), Observed rainfall correlation with the IOD and ENSO, respectively. (**c**) and (**d**), The same as (**a**) and (**b**), but from the multi-model ensemble mean (MMEM). Areas within the green contours in (**a**) and (**b**) represent regions where the correlation is statistically significant above the 95% confidence level. The significance is calculated using a two-tailed Student’s *t*-test. The green contours in (**c**) and (**d**) denote areas where the sign of the correlation is consistent over 80% of CMIP5 models. All maps were generated by NCL version 6.3.0 (http://dx.doi.org/10.5065/D6WD3XH5).

We focus on JJAS because these months see fast development of both ENSO and the IOD, and are the time when ISMR peaks. The impact on ISMR from the IOD and from ENSO tends to offset during JJAS, and this was documented using observations and model simulations^12^. For example, the 1997 El Niño event was strongest of the 20^th^ century, which alone would lead to an ISMR reduction, but a moderate ISMR increase occurred; this is because ENSO’s influence on ISMR was overwhelmed by the concurrent 1997 extreme positive IOD event^21^, usually considered as a secondary influence on the ISMR^13^. As such, in observations the correlation map between the IOD and the ISMR differs from its ENSO counterpart (Fig. 1a,b), despite the recent weakening of the ENSO-ISMR relationship^22^ and an increased frequency of central Pacific ENSO events^23, 24^. Rainfall responses to ENSO show a broadly consistent and significant region in the Indian subcontinent, i.e., a monsoonal rainfall reduction during El Niño (Fig. 1b). However, there is no significant rainfall response to IOD events (Fig. 1a), as the dominant and concurrent effect from ENSO suppresses the impact from IOD events.

**Figure 2:**
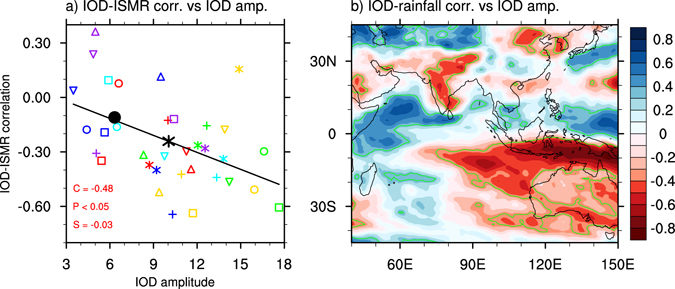
Inter-model simulations of present-day IOD properties. Inter-model variations in IOD amplitude (units: °C) versus variations in (**a**) IOD-ISMR correlation and (**b**) IOD-rainfall correlation in each grid point for the present-day climate. In (**a**), the linear regression line (black) with slope (S), correlation (C), and p-value (P) provided; the observed (MMEM) is indicated by a black circle (asterisk); and each model is represented by a colored symbol (see Fig. 3 for model names). The green contours in (**b**) denote areas where the correlation is statistically significant above the 95% confidence level. The significance is calculated using a two-tailed Student’s *t*-test. The plot and map were generated by NCL version 6.3.0 (http://dx.doi.org/10.5065/D6WD3XH5).

The ENSO-ISMR correlation has been reasonably simulated by climate models, and this is seen in the majority of models^25–27^. However, in terms of the MMEM, ENSO’s influence on the ISMR is stronger than the observations (Fig. 1b,d). The IOD-ISMR correlation pattern (Fig. 1c) resembles the ENSO-ISMR correlation pattern (Fig. 1d). This implies that there is an overly strong IOD-ISMR negative correlation in the present-day simulation; i.e., positive IODs tend to be associated with an ISMR reduction, more so in models. Figure 2a shows that models with greater IOD amplitude produce a greater IOD-ISMR negative correlation. The same analysis was conducted for each grid point with respect to models (Fig. 2b), and the result further confirms that models with a greater IOD amplitude tend to produce a greater IOD-ISMR negative correlation. This is seen in other IOD-affected regions, for example, negative correlations over the IOD eastern pole and southern Australia but positive correlations in the east African countries^19^. The result contradicts with the previous studies in which the IOD-ISMR correlation without ENSO influence should be positive; and the positive correlation should increase with an intensified IOD^12, 28^. This is not the case that the positive IOD-ISMR correlation is suppressed in models.

### ENSO and the overly large IOD-ISMR negative correlation

**Figure 3:**
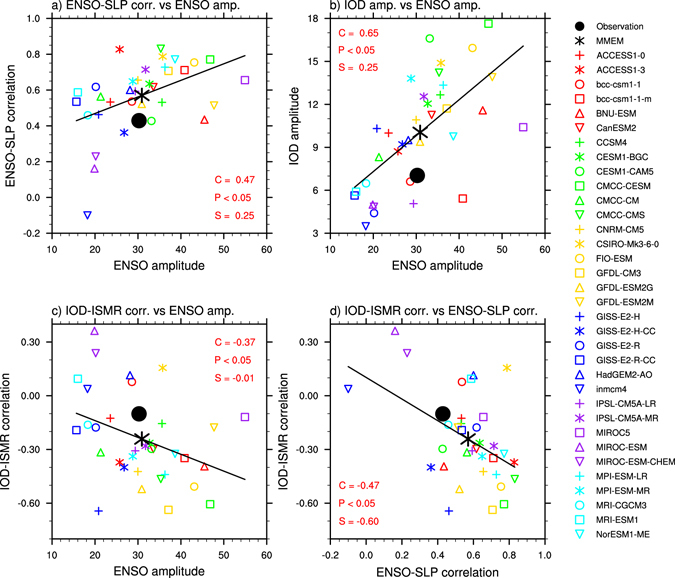
Inter-model relationships among ENSO, IOD and its atmospheric correlations. Inter-model relationship in present-day climate between (**a**) ENSO-SLP correlation and ENSO amplitude, (**b**) IOD amplitude and ENSO amplitude, (**c**) IOD-ISMR correlation and ENSO amplitude, and (**d**) IOD-ISMR correlation and ENSO-SLP correlation. The observations and MMEM are shown as the black circle and black asterisk, respectively. Each colored symbol represents a CMIP5 model. Solid line in each plot denotes the linear regression line with correlation coefficient (C), p-value (P) and regression coefficient (S) provided. A p-value smaller than 0.05 means statistical significance above the 95% confidence level. All plots were generated by NCL version 6.3.0 (http://dx.doi.org/10.5065/D6WD3XH5).

To date, it is not clear what causes the significant negative correlation in the simulated present-day climate, and how it is related to the model performance of ENSO simulation. Over the Indian subcontinent, the resemblance of Fig. 1c to d suggests that ENSO is a potential factor influencing the IOD-ISMR correlation. During El Niño, the SLP over the Indian subcontinent is stronger than normal, suppressing convection and resulting in rainfall reduction, and vice versa. Although the majority of CMIP5 models simulate such SLP response to ENSO, the ENSO-SLP correlations in most models are stronger than in observations (Fig. 3a). Further, models with a larger ENSO amplitude tend to produce a greater correlation, and the tendency is systematic, with the inter-model correlation (C = 0.47), statistically significant above the 95% confidence level. These overly strong SLP anomalies suppress development of any negative SLP anomalies, or positive rainfall anomalies, a concurrent positive IOD would induce.

Besides generating the overly large SLP anomalies over the Indian subcontinent, ENSO affects IOD properties such as amplitude. Previous studies have demonstrated that ENSO is a trigger of an IOD event^29–32^. During an El Niño event, the weakening of the Walker circulation generates easterly anomalies in the eastern and central equatorial Indian Ocean, lifting the central equatorial Indian Ocean and eastern Indian thermocline. This induces upwelling in the eastern Indian Ocean, leading to anomalous cooling in the surface water; the cooling contributes to an anomalous positive west-minus-east surface temperature gradient, which, in turn enhances the easterly anomalies in a Bjerknes positive feedback process^7, 33^. Indeed, there is an inter-model relationship showing that models with a greater ENSO amplitude systematically produce a greater IOD amplitude (Fig. 3b). However, for reasons yet to be understood, the Bjerknes-like positive feedback that operates in the eastern Indian Ocean is overly strong in models^34, 35^. Therefore, the response of the IOD to any forcing, including ENSO, is greater in models than in observations.

Given that ENSO overly influences IOD amplitude (Fig. 3b) and SLP anomalies over the Indian subcontinent (Fig. 3a), and given that the IOD amplitude is strongly associated with the IOD-ISMR negative correlation (Fig. 2a), it follows that models with a greater ENSO amplitude are associated with a greater IOD-ISMR negative correlation, and this is seen in Fig. 3c. In the majority of models, ENSO overly controls IOD amplitude because the simulated Bjerknes feedback in the eastern Indian Ocean is too strong. ENSO also enhances the IOD-ISMR negative correlation (Fig. 3c), or suppresses the IOD-ISMR positive correlation because the El Niño-induced positive SLP anomalies are overly strong (Fig. 3a). These overly strong ENSO-induced SLP anomalies suggest that ENSO *per se* contributes to the suppression of the IOD-ISMR positive correlation. This is confirmed from the inter-model relationship between the ENSO-SLP correlation and the IOD-ISMR correlation (Fig. 3d). Models with an overly strong ENSO-SLP positive correlation produce an overly strong IOD-ISMR negative correlation.

### Implications for future ISMR projection

**Figure 4:**
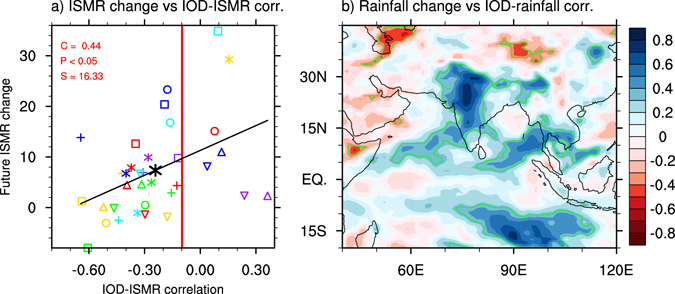
Inter-model relationship between future projections with present-day simulations. Inter-model variations in the present-day IOD-ISMR correlation versus variations in (**a**) future ISMR change (units: % per °C of global warming), and in (**b**) grid-point future rainfall change (units: % per °C of global warming) vs grid-point rainfall correlation with the IOD. In (**a**), the solid black line denotes the linear regression, and symbols for models are the same as in Fig. 3, and the red vertical line represents the observed IOD-ISMR correlation in present-day climate. Areas within green contours in (**b**) denote regions where the correlation is statistically significant above the 95% confidence level. The statistical significance is calculated using a two-tailed Student’s *t*-test. The plot and map were generated by NCL version 6.3.0 (http://dx.doi.org/10.5065/D6WD3XH5).

The aforementioned biases are large and common in CMIP5 models. Are they relevant to future projection of ISMR under greenhouse warming? If relevant, models that simulate a larger IOD-ISMR negative correlation should systematically produce a greater ISMR change in the future, and the inter-model relationship should be statistically significant. Indeed, the inter-model relationship between future ISMR changes and the present-day IOD-ISMR correlation shows that models with a greater IOD-ISMR negative correlation does tend to produce a smaller ISMR increase in the future, and the tendency is significant above the 95% confidence level (Fig. 4a). Such tendency is also seen in a correlation map between grid-point rainfall changes and the present-day grid-point IOD-rainfall correlation (Fig. 4b).

This result is consistent with the mean state change in the Indo-Pacific Ocean. Under greenhouse warming, the majority of models produce a warming pattern with a slower warming in the east than in the west Indian Ocean, namely a positive IOD-like warming pattern^35, 36^. However, this pattern is in part induced by the weakening of the Pacific Walker circulation^37^, which features a faster warming in the eastern than the western equatorial Pacific^38–40^. Thus, the inter-annual process in which a weakening Walker circulation leads to overly large positive SLP anomalies suppressing the IOD-ISMR positive correlation, also operates in the long-term timescale; in other words, the overly strong IOD-ISMR negative correlation on inter-annual time scales is not alleviated by the positive IOD-like long-term warming trend, because the latter is a consequence of weakening of the Pacific Walker circulation.

Therefore, the extent of the projected ISMR increase depends at least partially on how well the present-day IOD-ISMR correlation is simulated. In the present case, the projected ISMR increase is underestimated because of the bias of the overly large IOD-ISMR negative correlation. In other words, without this bias the projected ISMR increase would be greater. Here, we estimate the potential impact of the underestimation in the projected ISMR increase, based on the present-day IOD-ISMR correlation derived from observations and MMEM simulations. The difference between the future ISMR change derived from the MMEM IOD-ISMR correlation (black asterisk in Fig. 4a) and that from observations (intersection of red line and black line) is about 2.4% per °C of GW. Assuming a global warming of 4 °C in the next century, the total underestimated ISMR increase will be nearly 10% of climatological ISMR of the present-day climate. As 10% fluctuations of ISMR are considered as the threshold for drought and flood^1^, this magnitude of underestimation due to the bias in the IOD-ISMR correlation alone could have far reaching implications in terms of climate change adaptation options.

## Conclusions

We assessed the ISMR correlation with ENSO and the IOD in climate models and found that there is an overly strong IOD-ISMR negative correlation compared to observations. We demonstrated that the overly large IOD-ISMR negative correlation results from the exceedingly strong El Niño-induced positive SLP anomalies over the Indian subcontinent. This contributes to the suppression of an IOD-ISMR positive correlation. We showed that this bias in the present-day climate is relevant to future projection of ISMR, and would lead to an underestimation in the projected future ISMR increase. Our result suggests that the uncertainties in ISMR projection can be in part induced by the present-day simulation of ENSO, the IOD, their relationship, and their rainfall correlations. Therefore, improving model simulation of the IOD, ENSO, the feedback processes, and their relative importance in regional correlations is important for increasing our confidence in projected ISMR changes.

There are many avenues in which the present-study can be extended. Firstly, Indian summer monsoon winds can in turn affect the IOD development^41^ in a positive feedback. Whether models simulate this feedback and whether the realism of model simulation of this feedback contributes to the weak IOD-ISMR positive correlation need to be investigated. Secondly, a positive-IOD SST pattern promotes monsoonal cross-equatorial atmospheric flows conducive to increased ISMR^42^. It is not clear the overly-weak IOD-ISMR positive correlation in models is solely due to the overly-strong control by ENSO, or also due to poor simulations of other factors such as the IOD-induced cross-equatorial flows. Finally, we have not entered into the debate as to the relative importance of the dynamic and thermodynamic change to the projected ISMR increase^43–46^. Whether under greenhouse warming, a change in the IOD properties contributes to the relative importance needs to be assessed. Nevertheless, everything being equal, without the bias of the overly-weak IOD-ISMR positive correlation, the projected ISMR increase would be larger.

## Data and Methods

### Data

We analyse a suite of simulations from 34 models taking part in CMIP5, in which historical and representative concentration pathway (RCP) 8.5 scenarios are used to simulate the present-day and future climates respectively. Here, SST, precipitation, and sea level pressure (SLP) outputs from one ensemble member (r1i1p1) in each model are utilized (see Fig. 3 for a list of models). We define the second half of the 20^th^ century (1950–1999) as the present-day climate and the entire 21^st^ century (2000–2099) as the future climate. SST from the Hadley Centre Global Sea Ice and SST (HadISST1)^47^ and atmospheric circulation variables from the National Centers for Environmental Prediction–National Center for Atmospheric Research reanalysis (NCEP-NCAR Reanalysis)^48^ are used to provide an observational reference for the present-day (1950–1999) climate simulations.

### Methods

All data are bi-linearly interpolated onto a uniform 1° × 1° grid. Anomalies in present-day climate are referenced to the period of 1950–1999 and linearly detrended. The IOD is described through an Empirical Orthogonal Function (EOF) analysis on detrended SST anomalies in the tropical Indian Ocean domain (20°S-20°N, 40°E-120°E). In the EOF analysis, the IOD mode does not always come out as EOF1. For each model, we correlate the observed IOD pattern with each of the model EOF patterns. The EOF pattern that gives highest correlation is objectively identified the IOD mode in the model. The IOD index is defined as the associated time series, which, with the total variance of the EOF pattern scaled to unity^35^, represents intensity of the IOD mode in each model.

Similarly, the ENSO index is derived from EOF analysis over the tropical Pacific domain (20°S-20°N, 120°E-80°W)^20^. The ISMR index is the area-averaged rainfall anomalies over the Indian subcontinent (7°N-28°N, 70°E-90°E), and the SLP index is the area-averaged anomalies over the same domain. Future ISMR changes are expressed in terms of percentage change in present-day climatology per degree Celsius of global warming [GW; % (°C of GW)^−1^] to enhance comparability because models have vastly different climate sensitivities. Here, we use two metrics to benchmark the realism of ENSO and IOD simulations in models: (1) the amplitude of the IOD (ENSO) measured by the standard deviation of the IOD (ENSO) index; (2) the response of atmosphere circulation to the IOD (ENSO) defined as the correlation between atmospheric fields with the IOD (ENSO) index^28^.

### Seasonality

All calculations in this paper are performed on the JJAS period (June, July, August and September), the peak season of ISMR and the developing phase of ENSO and the IOD.

### Graphic software

All plots and maps were generated using:

The NCAR Command Language (Version 6.3.0) [Software]. (2016). Boulder, Colorado: UCAR/NCAR/CISL/TDD. http://dx.doi.org/10.5065/D6WD3XH5.
